# Parameters Optimization of Laser-Grinding Chain for Processing Groove of 2.5-Dimensional C/SiC Composites

**DOI:** 10.3390/ma16134761

**Published:** 2023-06-30

**Authors:** Guoyue Liu, Jian Wang, Bing Chen, Bing Guo, Hua Zhang, Zhaohui Wang

**Affiliations:** 1 Hunan Provincial Key Laboratory of High Efficiency and Precision Machining of Difficult-to-Cut Material, School of Mechanical Engineering, Hunan University of Science and Technology, Xiangtan 411201, China; guoyueliu2020@126.com (G.L.); jilinwang1996@163.com (J.W.); xyzh7@sina.com (H.Z.); 2 Hunan Taijia New Material Technology Co., Ltd., Changsha 410203, China; 3 Center for Precision Engineering, School of Mechatronics Engineering, Harbin Institute of Technology, Harbin 150001, China; guobing@hit.edu.cn; 4 College of Mechanical Automation, Wuhan University of Science and Technology, Wuhan 430081, China; zhwang@wust.edu.cn

**Keywords:** C/SiC composite, laser-grinding chain processing, groove feature, parameter optimization

## Abstract

Aiming at problems such as poor precision of laser processing C/SiC composites, low efficiency of grinding C/SiC composites, and serious wear of grinding wheel, a method of laser-grinding chain processing C/SiC composite groove was proposed in this paper. The method combined the high efficiency characteristic of laser ablating and the high precision characteristic of grinding. The relationship between laser processing parameters and the characteristics of ablative grooves was investigated, and the appropriate laser processing parameters were optimized, and then, to further improve the processing quality of the grooves, the grinding parameters optimization experiments of the grooves of C/SiC composites were carried out. The results showed that the C/SiC composites could be quickly removed by laser processing, and the grooves with relatively good size and shape accuracy could be obtained by laser parameters optimization experiments, but the side wall of the groove still had a certain inclination and the surface quality of the groove was yet poor; meanwhile, the size accuracy, shape accuracy, and surface quality of the grooves were greatly improved by further grinding. In addition, then, through the laser and grinding optimization experiments, the optimized parameters were obtained, where the laser power was 80 W, the scanning speed was 300 mm/s, the scanning frequency was 50 kHz, the scanning spacing was 25 μm, the depth of cut was 30 μm, the liner speed of wheel was 62.832 m/min, and the feed speed was 10 mm/min. With these parameters, the time used to process the groove with the laser-grinding chain was about 23/40 of the grinding process, and the quality of grooves could be guaranteed. Therefore, combined with the optimized process parameters, the laser-grinding chain processing scheme could be used to achieve high efficiency and precision grinding of C/SiC composite grooves.

## 1. Introduction

Carbon fiber composite material is a new type of material which is composed, of carbon fiber as reinforcement and resin, ceramic, and metal as matrix, through a special process [1,2]. The properties of the composite material are also different due to different matrix [3]. Among them, C/SiC composites not only possess the characteristics of high modulus, light weight, and good toughness of carbon fiber materials, but also possess the characteristics of high hardness, high fatigue resistance, and high temperature resistance of SiC materials [4,5,6,7]. C/SiC composites are listed as the preferred materials for high-performance friction materials and thermal protection structural parts of aerospace vehicles [8]. These composites are used in brake pads for high-end cars and planes, thermal protection systems, and thermal protection panels for aircraft, blades and nozzles for rocket engines, and substrates for space telescopes [9,10,11,12,13,14,15]. However, due to the high brittleness, wear resistance, and anisotropy of C/SiC composites, it is difficult to process with precision [16].

Laser processing is an efficient, non-contact, non-wear removal process, which can effectively avoid the defects of traditional processing methods, and has been used as the main technical means for processing carbon fiber ceramic matrix composites [17,18,19,20]. Liu et al. [21] carried out millisecond laser hole-making experiments on C/SiC composites and revealed the changes in the surface morphology of the recast layer inside the microporous walls and evolution of fiber ablation. Wang et al. [22] used the pulse laser to ablate C/SiC composites, and discovered that the edges of the sidewalls and bottoms were covered with an oxide layer composed of small white SiO_2_ particles: this was due to the rapid rise in temperature during the laser ablation process; meanwhile, the SiC was vaporized, and then, the gasified SiC was oxidized to white SiO2 particles, which covered the surface of the grooves of C/SiC composites. In order to suppress the ablative oxidation phenomenon of the processing surface, Zhai et al. [23] investigated the effect of laser processing parameters on the SiC/SiC processing morphology by femtosecond laser ablation experiments, and the results showed that the oxidation phenomenon became more and more obvious with increasing laser power, repetition frequency, and the number of scans, while the oxidation phenomenon gradually decreased with the increase in scanning speed. In order to obtain the circular hole depth and taper with good consistency, Liu et al. [24] used the femtosecond laser to conduct circular hole ablation experiments on SiC /SiC composites, and studied the influence of laser processing parameters on the characteristics of circular holes, the results showed that the depth of circular holes increased and the taper decreased with the increase in pulse energy, the increase in repetition frequency, and the decrease in scanning speed. In addition, nanosecond pulsed lasers can be used process C/SiC composites: Jiao et al. [25] conducted nanosecond pulsed laser ablation experiments on 2.5-dimensional C/SiC composites and discovered that ablation phenomena in the laser ablation area after nanosecond laser power and time reached a certain value, such as when ablation holes, resetting, fiber fracture, and terminal gas expansion occurred. Tong et al. [26] studied the ablation behavior of C/SiC composites by pulsed laser ablation, and the results showed that three ablation zones were observed on the ablation surface, the ablation center was acicular structure, a large number of spherical SiC particles were deposited in the transition zone, and the ablation edge area was covered by SiO_2_ layer. Pascale et al. [27] investigated the physical and chemical changes in Si and SiC–TiC–TiB_2_ composites after ultra-short laser ablation. The results indicated that fusing and resolidification may occur after laser ablation, and led to the index change in crystal structure, and some of the SiC transformed into Si during the experiment. To obtain the consistent width and depth of grooves, Jiao et al. [28] investigated the influence of laser process parameters on the ablation morphology, and discovered that the depth and width of grooves increased with the increase in laser power, and increasing the scanning speed and scanning frequency could reduce the extent of the jagged structure and improve the quality of the ablation surface. Chen et al. [29] explored the removal mechanism of nanosecond pulsed laser ablation of SiC/SiC composites by numerical simulation and experiment, and analyzed the effects of laser parameters on the ablation depth and heat-affected zone: the results showed that the ablation depth and heat-affected area depth increased as laser power, pulse width, pulse frequency, and scan spacing decreased, and the ablation depth and heat-affected zone depth first increased and then decreased with the increase in scanning speed.

Grinding has become a kind of high precision machining of various ceramic composite materials a technical means, but due to the characteristics of the material, the machining process will produce serious tool wear, machining surface integrity is poor, so efficient and high-precision machining of ceramic composite materials is still a huge technical challenge [30,31,32]. At present, domestic and foreign scholars’ research on the grinding of carbon fiber ceramic matrix composites mainly focused on the effects of processing parameters, fiber direction, and ultrasonic vibration assistance on grinding force, processing quality, and material grinding removal mechanism, while the research on grinding wheel wear in the process of carbon fiber ceramic matrix composites is relatively limited [17]. Xing et al. [33] analyzed the influence of different processing parameters on tool wear by brazing diamond tools to process C/C-SiC ceramic matrix composite materials, the tool wear was mainly concentrated in the outer ring where the liner speed of wheel was relatively high, and the tool wear was lighter under the condition of low liner speed of wheel. At the same time, the high grinding temperature caused adhesive wear when the feed speed was excessive. Chen et al. [34] used the brazed diamond liner drill to process SiC_f_/SiC composites with ultrasonic vibration assistance; by observing the abrasive wear morphologies before and after grinding SiC_f_/SiC composites, it could be seen that the abrasive wear characteristics of the end face after the liner drill failure are mainly wear platform and macro crushing, and the wear of side wear was serious with the increase in the distance between side wear and the end face. Gu [35] used a brazed diamond sleeve drill with a grinding particle size of 40# to make holes in C/SiC composites with rotary ultrasonic-assisted processing; the results showed that the abrasive wear characteristics of the tool face and side were abrasive shedding, wear platform, and macro breakage. However, due to the tool wear speed being faster, the hole-making process was divided into the initial processed stage, smooth processed stage, and processed failure stage, and the number of holes stably processed by the tool was about 40. Weinert et al. [36] used an electroplated diamond liner drill with grooves to make holes for C/C-SiC and found that the degree of tool wear was negatively correlated with the axial length of the tool. When the abrasive particles were completely worn, the tool matrix directly participated in grinding, and cracks appeared in the groove of the matrix surface due to excessive stress, the cracks expanded irregularly along the direction of the tool and finally made the tool completely ineffective. Wang et al. [37] carried out comparative experiments on C/SiC composite grooves processed by laser and grinding, and the results showed that the grooves processed by laser were inclined, had uneven bottom surface morphology, and ablative oxidation defects: the groove was processed for a time of 80 s. The surface quality of the grooves after grinding was improved to some extent; however, due to the severe wear of the grinding wheel, the shape accuracy of the grooves after grinding was extremely poor, and the processing time of the grooves of the same size was 8000 s, the processing efficiency was low. Bertsche et al. [38] carried out tests of rotary ultrasonic grinding and ordinary grinding of ceramic matrix composites, and the results show that compared with ordinary grinding of ceramic matrix composites, rotary ultrasonic grinding can significantly reduce the machining cutting force and tool wear.

Summing up, the current research on laser ablation of carbon fiber ceramic matrix composites mainly focuses on surface morphology, material ablation removal mechanism, and the influence of processing parameters on the characteristics of blind holes and grooves, there are few reports on ablation oxidation defects and slope removal after laser ablation. Moreover, the better quality of carbon fiber ceramic matrix composites could be obtained by grinding, but the processing efficiency was low, and the grinding wheel wear was serious. In order to solve the problems existing in laser and grinding processing of 2.5-dimensional C/SiC composite grooves, the laser-grinding chain processing experiment of 2.5-dimensional C/SiC composite grooves was carried out in this paper, so as to obtain efficient and high-quality processing of 2.5-dimensional C/SiC composite grooves. The relationship between the scanning frequency and scanning spacing and the dimension characteristics for the groove of the ablation C/SiC composites were investigated, and the appropriate laser processing parameters were optimized; on this basis, the ablative oxide layer and sidewall taper after laser processing were removed by grinding. To further improve the processing quality of the grooves, the grinding parameters experiment was carried out to improve the quality of the grooves for 2.5-dimensional C/SiC composites, and the appropriate grinding parameters were selected by performing range analysis, response curve analysis, and variance analysis on surface roughness, which laid a scientific foundation and technical route for the processing of 2.5-dimensional C/SiC composite grooves.

## 2. Experimental Material and Processed Equipment

**Figure 1:**
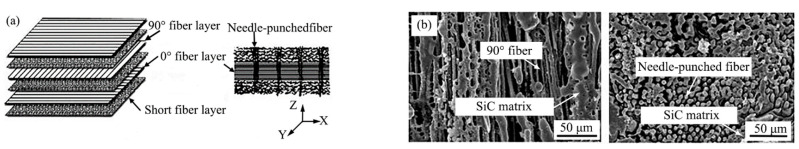
Fiber arrangement pattern and microstructure (**a**) Fiber arrangement pattern, (**b**) Microstructure.

**Figure 2:**
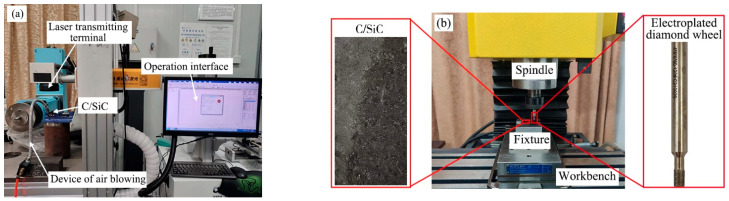
Processing equipment diagram (**a**) Laser process equipment, (**b**) Grinding equipment.

C/SiC composite workpieces used in this experiment were manufactured by the chemical vapor infiltration (CVI) method of Gong Yi Van-Research Innovation Composite Material Co. Ltd. (Kongyi, China), the workpiece size was 30 mm × 15 mm × 9.8 mm, the internal structure consists of carbon fibers, SiC matrix, and randomly distributed pores, the volume fraction of carbon fibers was 40–50%, the mass fraction of carbon fibers was 27–36%: the fiber arrangement and microstructure are shown in Figure 1. The fiber arrangement and microstructure are shown in Figure 1. The tool selected in this paper was an electroplated diamond grinding wheel, which was prepared by Zhengzhou Abrasives Grinding Research Institute Co., Ltd. (Zhengzhou, China), and its specific parameters were as follows: particle size of 120 #, abrasive concentration of 100%, wheel diameter of 4 mm, tool holder diameter of 6 mm, and grinding wheel handle length of 60 mm. The experiments were processed by a nanosecond laser marking machine(Shenzhen Orena Laser Equipment Co., Ltd., Shenzhen, China) and a small CNC milling machine JX300 (Yornew Automation Equipment Co., Ltd., Foshan, China) for C/SiC composites, as shown in Figure 2. The experiments were processed by a nanosecond laser marking machine and a small CNC milling machine JX300 for C/SiC composites, as shown in Figure 2.

## 3. Experimental Scheme

The feasibility of laser-grinding chain processing of a 2.5-dimensional C/SiC composite groove was preliminarily explored, and compared with grinding processing, the surface roughness of the groove after laser-grinding chain processing was improved by 1.27–1.96 times, the processing time was about 1/3 of that of grinding processing, and the grinding wheel wear was relatively light, but it still took about 2418 s to process a groove with the size of 10 × 6 × 2 mm; the processing efficiency was still low, because the low dimensional accuracy, shape accuracy, and surface quality of the laser-ablated C/SiC composite grooves would increase the removal amount of subsequent grinding, and thus decrease the grinding efficiency [39]. Therefore, the process optimization of laser-grinding chain processing of 2.5-dimensional C/SiC composite groove was studied, the relationship between nanosecond pulsed laser processing parameters and groove dimensional characteristics and processing quality was analyzed, and the grinding parameters optimization experiments were conducted, and then, the processing efficiency and accuracy of 2.5-dimensional C/SiC composite could be improved simultaneously.

### 3.1. Design of Laser Processing Experiments

The influence of laser power and scanning times on the quality of ablated blind holes was studied by the subject group in the early stage, and the results showed that the ablative quality was better when the laser power (*P*) was 80 W and scanning speed (*v*) was 300 mm/s [40]. Therefore, the laser power, the scanning speed, and the scanning times (*N*) were, respectively, set to 80 W, 300 mm/s, and 20 times chosen in this experiment, and the influence of scanning frequency (*f*) and scanning spacing (*l*_s_) on the groove characteristics and ablation quality were investigated by the single-factor controlled variable method, the experimental parameters are shown in Table 1.

**Figure 3:**
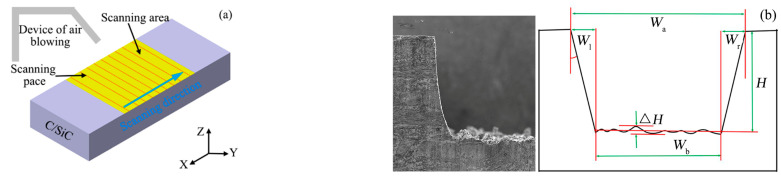
Schematic diagram of groove scanning trajectory and evaluation characteristics of grooves (**a**) Schematic diagram of groove scanning trajectory. (**b**) Evaluation characteristics of grooves.

The filling scanning mode adopted for nanosecond pulsed laser ablation of C/SiC composite grooves was parallel filling scanning, the filled rectangle size was 15 mm × 6 mm, the air-blowing device was installed above one end of the filled rectangle long axis, and the distance between the air outlet and the workpiece was about 15 mm, the schematic diagram of beam scanning trajectory is shown in Figure 3a. Firstly, with the laser scanning frequency as the variable, the laser power, scanning speed, scanning times, and scanning spacing were, respectively, set to 80 W, 300 mm/s, 20 times, and 20 μm, and the scanning frequency was set to 50 kHz, the laser beam scanned from the leftmost track of the groove to the rightmost side of the groove, the laser focus was moved down 50 μm along the Z direction, and the next layer was scanned, and scanning was repeated 20 times. After this group of experiments was completed, the scanning frequency was, respectively, set to 100 kHz, 150 kHz, 200 kHz, 250 kHz, and 300 kHz, and the procedure was same as above. Then, with the scanning spacing as the variable, the laser power, scanning speed, scanning times, and scanning frequency were, respectively, set to 80 W, 300 mm/s, 20 times, and 200 kHz, and the scanning spacing was, respectively, selected as 5 μm, 10 μm, 15 μm, 20 μm, 25 μm, and 30 μm, and the above processing process was repeated.

The important indicators which could express the efficiency and quality of nanosecond laser ablation groove in 2.5-dimensional C/SiC composites include the width of the upper surface of the groove (*W*_a_), the width of the lower surface of the groove (*W*_b_), the average depth of the grooves (*H*), the taper of the groove side wall (*θ*), and the depression depth of the groove bottom(Δ*H*) [41], as shown in Figure 3b, where the most influential factors were the taper of the groove side wall and the depression depth of the groove bottom.

**Figure 4:**
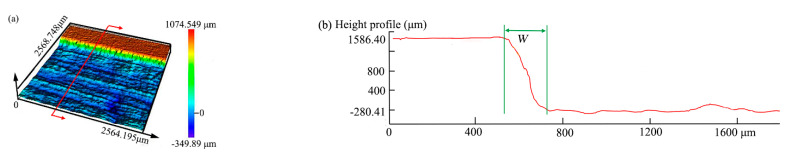
Three-dimensional surface morphology and contour morphology of C/SiC composites obtained by confocal microscopy (**a**) Three-dimensional surface morphology. (**b**) Contour map of C/SiC composites.

After the C/SiC composite grooves were ablated by the nanosecond pulsed laser, the width of the groove top and bottom were measured by the electron microscope, the average depth and the depression depth of the groove bottom were measured by the confocal microscopy, and the taper of the groove sidewall was calculated according to the Formula (3). The measurement result of the groove from the confocal microscope after 20 times scanning is shown in Figure 4a, the cross-section profile obtained along the 1/4 length of groove is shown in Figure 4b (two positions were selected), measurements with confocal microscopy were also taken at 1/2 (two positions) and 3/4 (two positions) of the groove length. Then, Formulas (1)–(3) were used to calculate the depression depth of the groove bottom, the average depth and the taper of the groove side wall at 1/4, 1/2, and 3/4. Finally, the average of several times was taken as the final result.

The depression depth of groove bottom (Δ*H*) can be calculated as:

(1) $$\Delta H=\frac{\sum_{i=1}^{12}\left(H_{i}-h_{i}\right)}{12}$$

where *H*_i_ is the maximum values of the groove depth at the cross-section, *h*_i_ is the minimum values of the groove depth.

The formula for calculating the average depth of the groove (*H*) could be described as:

(2) $$H=\frac{\sum_{k=1}^{12}\left(H_{k}\right)}{12}$$

where *H*_k_ is the average depth of the groove at the cross-section.

The taper of the groove sidewall (*θ*) could be calculated as:

(3) $$\theta =\arctan(\frac{800\;W_{}}{7H})$$

where *W* is the measurement under the confocal microscope, *H* is the average depth of the groove.

#### Experiment Design for Grinding Parameters Optimization in Laser-Grinding Chain Process

Firstly, the optimal grooves of C/SiC composites were ablated by nanosecond pulsed laser using the optimized laser processing parameters, these optimal grooves were the base grooves used in the grinding parameters optimization experiment. In addition, then, three-factor and three-level orthogonal grinding experiments were carried out on the ablation grooves. By reviewing a large number of relevant references, three levels of depth of cut were 30 μm, 50 μm, and 70 μm, three levels of wheel liner speed were 37.7 m/min, 50.3 m/min, and 60.8 m/min, and three levels of feed rates were 10 mm/min, 20 mm/min, and 30 mm/min were selected, and the orthogonal design parameters are shown in Table 2.

**Figure 5:**
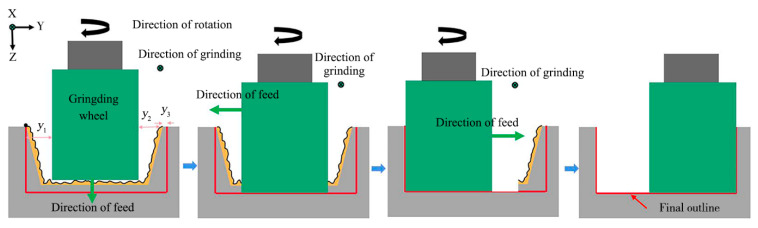
Grinding process.

The scheme of grinding processing was designed in Figure 5. Firstly, the grinding wheel was moved along the Y+ direction to the center of the ablation groove, and moved along the Z+ direction to contact the groove bottom, and then, the bottom of the grinding wheel was tooled at the center of the ablation groove bottom. Secondly, the grinding wheel was moved to the end face of the groove along the X- direction, the processing allowance and ablative oxide layer on the surface of the groove was removed by grinding wheel outwards and inwards from the workpiece end face, and, according to the calculation, the depth of the groove reached the ideal size when grinding n times. Then, the grinding wheel was moved to Y- with cutting depth of 50 μm each time, and the groove was ground from the workpiece end face from outside to inside, the sidewall taper of the left groove was removed after grinding *y*_1_ to the left. Finally, the grinding wheel was moved *y*_1_ mm along the Y+ direction, it began grinding to the right side of the groove, the right-side wall taper of the groove was removed when ground *y*_2_ to the right, and the width of the groove was qualified when ground *y*_3_ to the right.

The calculation time of C/SiC composite grooves processed by laser-grinding chain is shown in Equation (4):

(4) $$T_{1}=N\left(\frac{B}{D}-1\right)\frac{L}{v}+\frac{L}{v_{f}}\left(6+\frac{y_{1}+y_{2}+y_{3}}{a_{p}}\right)$$

where *B* is the width of the groove; *L* is the length of the groove; *D* is the laser scanning spacing; *v* is the scanning speed; *N* is the scanning times; *v*_f_ is the feed speed, *a*_p_ is the depth of cut, *y*_1_ + *y*_2_ + *y*_3_ = 2 mm.

The calculation of the time used for grinding the grooves of C/SiC composites is shown in Equation (5):

(5) $$T_{2}=\frac{L}{v_{f}}\left(\frac{2000+2000}{a_{p}}\right)$$

where: *L* is the length of the groove, *v*_f_ is the feed speed, and *a*_p_ is the depth of cut.

When the laser power was 80 W, the scanning speed was 300 mm/s, the scanning frequency was 200 kHz, the scanning spacing was 25 μm, the scanning time was 20, the depth of cut was 50 μm, the liner speed of the wheel was 62.832 m/min, and the feed speed was 20 mm/min, according to Formulas (4) and (5), the time used for processing grooves (15 mm × 6 mm × 2 mm) of the two schemes were theoretically calculated, the time used for laser-grinding chain processing was 1380 s, and the time used for grinding only was 2400 s: the calculated time only includes the processing time, and the auxiliary time was not calculated. Therefore, the laser-grinding chain processing time of C/SiC composite grooves was about 23/40 times that of grinding; it was proved that the laser-grinding chain method for processing groove of C/SiC composites had better efficiency.

## 4. Results and Discussion

### 4.1. Effect of Scanning Frequency and Scanning Spacing on the Macroscopic Morphology of the Groove Bottom Surface

**Figure 6:**
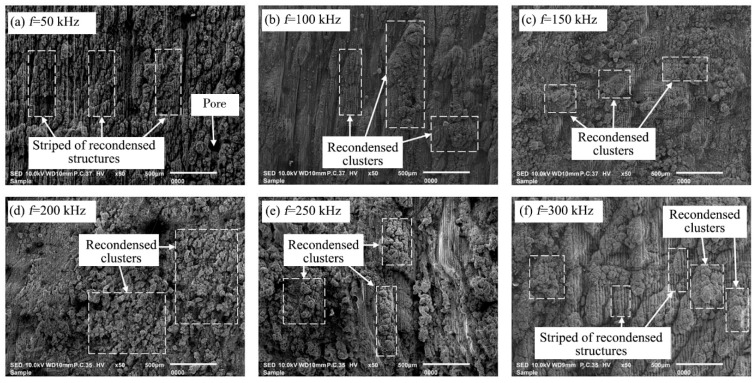
SEM morphology of the groove at different scanning frequency.

The bottom surface morphology of the 2.5-dimensional C/SiC composite grooves after nanosecond laser ablation not only reflects the quality of the groove, but also affects the efficiency of the subsequent finishing process. Therefore, the effect of scanning frequency on the surface morphology of the groove bottom after processing was investigated with the laser power of 80 W, scanning speed of 300 mm/s, scanning times of 20, and scanning spacing of 20 μm, as shown in Figure 6. From Figure 6, the carbon fiber and SiC matrix material on the bottom surface of the grooves were covered by recondensed material after C/SiC composites ablated. When the scanning frequency was 50 kHz, a large number of regular striped of recondensed structures and pores could be observed on the bottom surface of the grooves, which was because when the scanning frequency was low, the pulse overlap rate was small, the overlap area was small, and the material could be removed only when the pulse energy density was higher than the ablation threshold of the material; therefore, the striped recondensed structures could be found on the bottom surface of the grooves [40]. With the increase in scanning frequency, the striped recondensed structures gradually decreased or even disappeared, and the irregular recondensed material started to gather together and appear in the form of recondensed clusters, which was due to the increase in pulse overlap area with the increase in scanning frequency, the energy density per unit area increased, and the material was removed by uniform ablation [16]. When the scanning frequency increased to 300 kHz, the striped recondensed structures reappeared and formed the groove bottom surface morphology together with the recombination mass. In addition, it could also be seen that when the scanning frequency was 50 kHz, the surface topography was better than the surface topography after processing with other parameters, which was conducive to reducing the removal amount of subsequent precision grinding and thus improving the processing efficiency.

**Figure 7:**
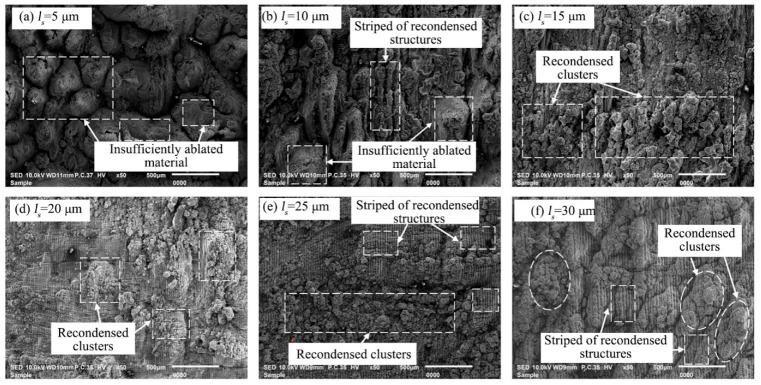
SEM morphology of the groove at different scanning spacing.

The influence of scanning spacing on the surface morphology of the groove bottom after nanosecond pulsed laser processing was studied when the laser power was 80 W, the scanning speed was 300 mm/s, the scanning times was 20, and the scanning frequency was 50 kHz, as shown in Figure 7. When the scanning spacing was 5 μm, it was obviously found that the C/SiC composite at the groove bottom was not fully ablated, and the material was unevenly removed and covered by recondensed material, which was because the scanning spacing was too small, the adjacent pulsed laser spots would overlap, and the high laser energy in the overlapping part made more material be removed [41]. The phenomenon of insufficient ablation of the material decreased as the scanning spacing increased, when the scanning spacing was 10 μm, the striped recondensed structures and insufficient ablation of the material could be observed on the bottom surface of the grooves, when the scanning spacing was increased to 15 μm, the phenomenon of insufficient ablation of the material was not observed, and the recondensed material was distributed randomly at the groove bottoms. As the scanning spacing continued to increase, the recondensed material began to accumulate, and the recondensed clusters and striped recondensed structures could be observed at the groove bottom: this was because when the scanning spacing was too large, the ablation spatters and evaporated material would repeatedly fall on the groove surface when the material was removed by ablation before and after the adjacent laser lines, making the recondensed material accumulate and appear on the groove surface in the form of recondensed clusters [26]. In addition, when the scanning spacing was 25 μm, the depth values between the raised and recessed parts of the surface at the groove bottoms were significantly smaller than the depth values between the concave and convex parts of the surface obtained by other parameters, resulting in less removal material and fewer grinding times for the subsequent precision grinding process, which achieved the purpose of improving machining efficiency.

### 4.2. Effect of Laser Processing on the Microscopic Morphology of the Groove Bottom

**Figure 8:**
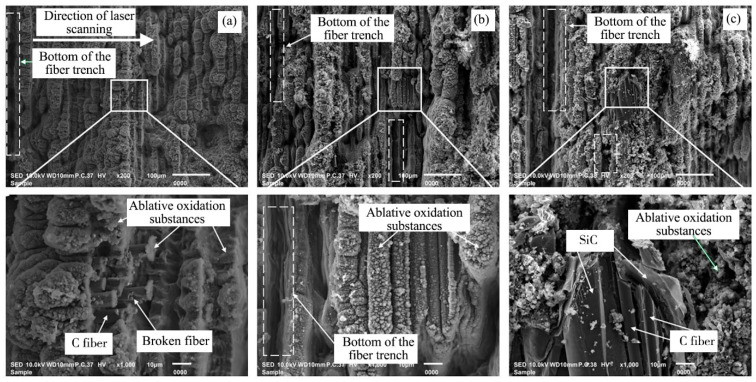
Microscopic morphology of the groove bottom after laser ablation. (**a**) Transverse fiber region, (**b**) Longitudinal fibrous region, (**c**) SiC matrix region.

The microscopic morphology of 2.5-dimensional C/SiC composite grooves after laser ablation was investigated with the laser power of 80 W, scanning speed of 300 mm/s, scanning spacing of 20 μm, scanning times of 20, and scanning frequency of 50 kHz, as shown in Figure 8. As can be seen from Figure 8a,b, a large number of powder, loose, and porous ablative oxidation substances appeared on the bottom surface of the ablation groove, the carbon fiber and silicon carbide matrix were almost covered, with only a few fibers and matrix materials leaking out, and the trench bottom of fiber could be found on the bottom surface of grooves, and it was hypothesized that the appearance of this structure was caused by two factors: firstly, the thermal diffusion rate inside the material was not uniform, and the conduction rate along the fiber direction was faster than that in other directions [42]; secondly, when ablating, the diffuse reflection was produced on the surface of the processed material so that less carbon fiber or matrix material received more energy, and thus more material was removed [43]. The same phenomenon was found in Figure 8c, where the bottom of the fiber trench could be observed, and the carbon fiber and silicon carbide matrix were almost covered, but some of the transverse fibers were found to be barely leaking out from the local magnified view, and the fibers and silicon carbide matrix were not uniformly removed at the same time, this was due to the different properties of carbon fiber and silicon carbide matrix materials, the sublimation temperature of SiC matrix was 2700 °C, while the sublimation temperature of carbon fiber was 3550 °C, which was higher than that of SiC, therefore, the carbon fiber was in an incomplete sublimation state and the ablation rate of SiC matrix was faster than that of C fiber [44]. In addition, fiber fragmentation was observed away from the fiber breaks, which was due to the different ablation properties of the various carbon fiber sections [21].

**Figure 9:**
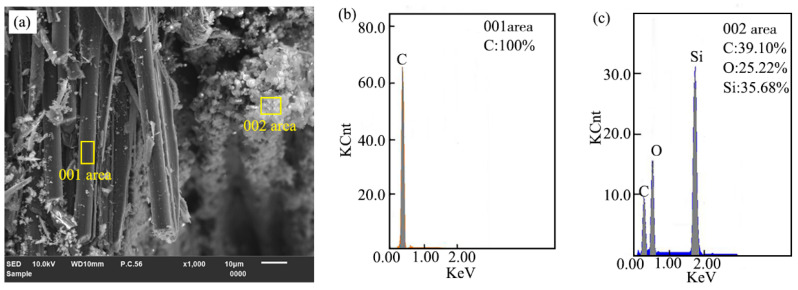
Detection of EDS components at the groove bottom ablation: (**a**) Micromorphology, (**b**) 001 area, (**c**) 002 area.

In order to confirm the composition of material adhering on the groove surface after laser ablation of 2.5-dimensional C/SiC composites, the EDS composition analysis was performed at random detection locations in the bare-leakage carbon fiber and material covered areas. As shown in Figure 9, only carbon elements were detected on the bare leaking carbon fiber surface, while C, O, and Si elements were detected in the material covered area, where the percentages of C, O, and Si contents is 39.10%, 25.22%, and 35.68%, respectively. The results indicated that the composition of substances adhering to the C/SiC composites ablation groove surface were silicon oxygen compounds and silicon carbide, this was because C/SiC composites underwent a series of physicochemical reactions under the action of high local temperature for the laser beam, with the accumulation of heat and the increase in temperature, the carbon fiber reached its sublimation temperature and formed carbon vapor, and the part of silicon carbide matrix reached its decomposition and sublimation temperature, and the cracked Si (g) was oxidized to generate a large amount of hot SiO_2_ gas and a small amount of SiO (g), which was vaporized and splashed out of the groove surface, and as the temperature decreased, the groove surface was covered with gaseous SiO_2_ and SiO in the form of smoke dust [45].

**Figure 10:**
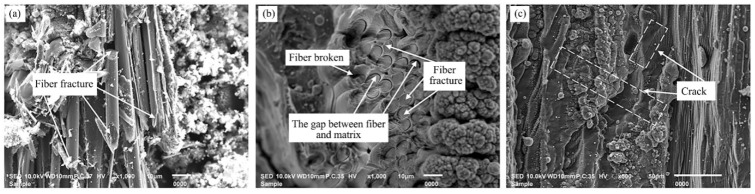
Microstructure of the ablative groove bottom in different regions: (**a**) the longitudinal fiber region, (**b**) the needled fiber region, (**c**) the SiC region.

Figure 10 shows the microscopic morphology of the longitudinal fiber region, the needled fiber region and SiC region at the bottom of the ablation groove. From the longitudinal fiber region after laser ablation in Figure 10a, the clear carbon fiber fractures could be observed, the fibers and matrix were not removed at the same time, the lengths of exposed longitudinal fibers were different, and the fibers all showed the state of being mitered. In addition, as can be seen from the needle-punched fiber region after laser ablation in Figure 10b, compared with the longitudinal fiber area after laser ablation, only a very small portion of the fibers were broken, the needle-punched fiber fracture was almost flush with the SiC substrate material, and the fiber fracture surface was flat, indicating that the needle-punched fibers were removed simultaneously with the matrix; meanwhile, the gap between fiber and matrix was found. From Figure 10c, it can be seen that cracks appeared on the surface of the SiC matrix, which was because of the Gaussian distribution of laser energy, with high energy in the center of the spot and low energy at the edges, and the surface temperature of the central irradiation area raised rapidly, resulting in the material in the center of the ablation area being melted, while the material at the edges of the ablation zone was not yet melted, resulting in a strong thermal stress concentration, and when the stress accumulated to a certain limit, the surface of the SiC substrate cracked or even the surface broke off [46].

### 4.3. Influence of Laser Scanning Frequency and Scanning Spacing on Groove Characteristics

**Figure 11:**
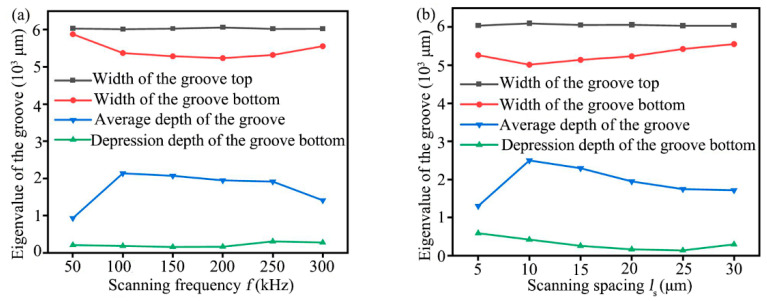
Relationship between different laser processing parameters and the eigenvalue of groove (**a**) Scanning frequency, (**b**) Scanning spacing.

**Figure 12:**
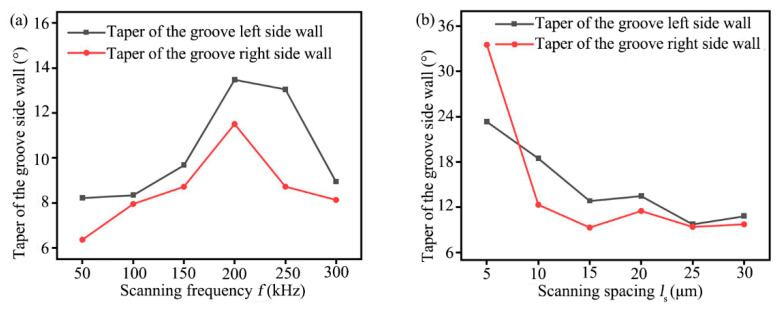
Relationship between different laser processing parameters and the taper of groove side wall (**a**) Scanning frequency, (**b**) Scanning spacing.

Laser processing was widely used for C/SiC composite materials due to its high efficiency and non-contact characteristics; however, the laser energy was distributed normally, resulting in a deviation of dimensional accuracy between the actual processing groove and the ideal processing groove being large [39]. To improve the dimensional accuracy, reduce the processing amount and grinding path of subsequent precision grinding, the effects of different scanning frequency and scanning spacing on the width of the groove top and bottom, the average depth of the groove, the depression depth of the groove bottom, and the taper of grooves side wall were studied, as shown in Figure 11 and Figure 12.

When the laser power was 80 W, the scanning speed was 300 mm/s, the scanning spacing was 20 μm, and the scanning times were 20, the effects of different scanning frequency on the width of the groove top and bottom, the average depth, and the depression depth were investigated, as shown in Figure 11a. According to Figure 11a, the width of the groove top hardly changed as frequency increased, but the actual processed width of the groove top was slightly larger than the standard groove size; the width of the groove bottom decreased first and then increased with the increased frequency. Meanwhile, the average depth of the grooves increased sharply to 2141.022 μm when the scanning frequency was increased from 50 kHz to 100 kHz, and then, as the scanning frequency was increased further, it changed little and showed a slightly decreasing trend: the former was due to the increase in the frequency of laser spot with the increase in scanning frequency, at the same time, the number of times of laser spot ablation on the workpiece surface increases, and the amount of material removal increases [28]; the latter was because as the scanning frequency continues to increase, the number of pulses increased, and the energy obtained by each pulse was small, when the pulse energy density was lower than the ablation threshold of the material, the material was not removed by ablation [39]. Moreover, the depression depth of the groove bottom decreased first and then increased with the increased scanning frequency, and the minimum value of the depression depth was 160.447 μm when the frequency was 200 kHz.

When the laser power was 80 W, the scanning speed was 300 mm/s, the scanning frequency was 50 kHz, and the scanning times were 20, the effects of different scanning spacing on the width of the groove top and bottom, the average depth, and the depression depth were investigated, as shown in Figure 11b. When the laser scanning spacing increased from 5 μm to 30 μm, the width of the groove top hardly changed, and the actual width of the groove top obtained was slightly larger than 6 mm. The width of the groove bottom decreased first and then increased, and the width of the groove bottom was less than 6 mm. Meanwhile, the average depth of the grooves increased first and then decreased with the increased scanning spacing, and when the scanning spacing was 10 μm, the average depth of the grooves reached the maximum value of 2501.528 μm. Moreover, the depression depth decreased first and then increased with the increased scanning spacing, and the minimum value of 141.915 μm was reached when the scanning spacing was 25 μm.

The taper of the ablation grooves side wall with different laser scanning frequency and scanning spacing can be calculated by Equation (3), as shown in Figure 12. From Figure 12a, it can be seen that when the scanning frequency increased from 50 kHz to 300 kHz, the taper of the groove side wall increased first and then decreased with the increased scanning frequency, and the taper of the groove left side wall was larger than that of the right side wall, and when the scanning frequency was 50 kHz, the taper of groove both left and right sidewall were the minimum value. From Figure 12b, it can be seen that when the scanning spacing was 25 μm, the taper of the groove left side wall reached the minimum value of 9.3172° and the taper of the groove right side wall reached the minimum value of 9.3943°. Mostly, the taper of the groove left side wall was larger than the taper of groove right side wall in the experiment, it was because of the accumulation of laser heat when ablating from left to right, and the temperature was low when the ablation began at the left, and the temperature was high when the ablation process reached the right [24].

In summary, taking into account size accuracy, shape accuracy, and surface morphology, and the influence of these faction the subsequent grinding allowance, the laser power of 80 W, the scanning speed of 300 mm/s, the scanning frequency of 50 kHz, and the scanning spacing of 25 μm were selected to use ablation before grinding. On these parameters, under the conditions of the laser processing parameters, the grooves with high processing efficiency and better processing quality can be obtained, but the ablative oxide layer and sidewall taper still exist after laser processing. To further improve the processing quality of the grooves, the grinding experiments were carried out in the groove after laser ablation, and the experiment was carried out to optimize the grinding parameters of the grooves of C/SiC composites by laser-grinding chain processing.

### 4.4. Surface Roughness Analysis of C/SiC Composite Groove by the Laser-Grinding Chain Processing

The grooves with relatively high processing accuracy and efficiency could be obtained through the laser parameter optimization experiment, but the processing quality of the groove was still poor. In order to further improve the processing quality of the grooves after laser ablation, the grinding parameter optimization experiment of C/SiC composite grooves was carried out, and grooves of C/SiC composites with high efficiency and good quality were obtained by range analysis and factor response analysis with different grinding parameters.

The range analysis table of the surface roughness *S*_a_ is shown in Table 3. It can be seen from Table 3 that the influence of factor A (depth of cut *a*_p_) on the surface roughness *S*_a_ was greatest, the influence of factor B (liner speed of wheel *v*_s_) was the second, and the influence of factor C (feed speed *v*_f_) was least, and it can be concluded that the combination of *A*_1_*B*_3_*C*_1_ was the optimal grinding parameters for C/SiC composite grooves in laser-grinding chain processing. In other words, when the depth of cut was 30 μm, the liner speed of wheel was 62.832 m/min, the feed speed was 10 mm/min, the surface roughness of the C/SiC composite grooves was the minimum, and the surface quality was the best.

**Figure 13:**
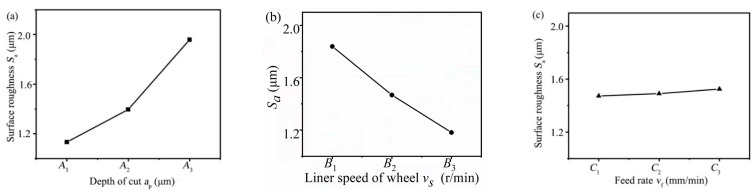
Response curve of the surface roughness *S*_a_. (**a**) Depth of cut, (**b**) Liner speed of wheel, (**c**) Feed rate.

The response curve of the surface roughness *S*_a_ is shown in Figure 13. The abscissa was different processing levels, and the ordinate was the mean value of surface roughness *S*_a_ with the same factor and different levels. As can be seen from the response curve of *S*_a_ in Figure 13, the surface roughness *S*_a_ both increased with the increased depth of cut *a*_p_ and feed rate *v*_f_, but in different ways, the former increased sharply while the latter increased slowly, and the surface roughness *S*_a_ was negatively correlated with liner speed of wheel *v*_s_.

### 4.5. Analysis of C/SiC Composites Grooves Quality

**Figure 14:**
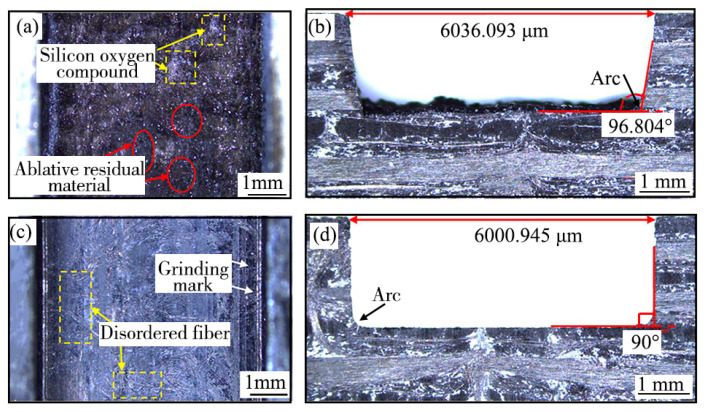
Macroscopic morphologies of the bottom and end surfaces of the grooves after different processing methods: (**a**,**b**) laser; (**c**,**d**) laser-grinding chain.

The macroscopic morphologies of the bottom and end cross section of the processed C/SiC composites grooves are shown in Figure 14. As shown in Figure 14a,c the bottom of the ablation C/SiC composite groove was uneven, the random distribution of white bright silicon oxygen compound and black ablative residual material could be observed on the bottom, while the bottom of the C/SiC composite groove after laser-grinding chain processing was flat, the white bright silicones and the black ablative residues were completely removed, and some disordered fibers were observed at the groove bottom, this was presumably because disordered fiber layer was ground at the end of grinding, and the fibers in different fiber areas were distributed at arbitrary angles in the disordered fiber layer. In addition, some deep grinding marks were formed on the processed surface of the groove bottom [47]. As shown in Figure 14b,d, the angle between the bottom and the sidewall of the ablation groove was 96.804°, and the actual width of the groove top was 6036.093 μm, which was larger than the standard groove size of 6000 μm. Moreover, the uneven surface could be observed on the groove bottom. However, the angle between the bottom and the sidewall of the groove by the laser-grinding chain processing was about 90°, and the actual width of the groove top was 6000.945 μm, which was approximately equal to the ideal groove size, the flatness and dimensional accuracy of the groove were obviously better than that of the ablation groove, besides, only the arc which affecting the shape accuracy of groove appeared at the corner of the side wall and bottom of the groove, this was because the grinding abrasives and matrix materials were subjected to greater grinding force and more grinding heat at the junction of the bottom surface and side wall of the grinding wheel, and then, the abrasives and matrix materials were easy to be worn, resulting in the materials at the junction of the bottom and side wall of the groove could not be fully removed [37].

**Figure 15:**
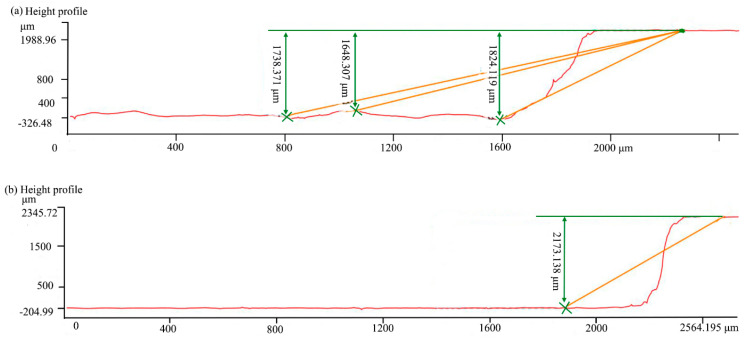
Confocal profile of the groove with different processing methods (**a**) laser; (**b**) laser-grinding chain.

Figure 14 only analyzes the macroscopic morphology of the end surfaces of the grooves by laser and laser-grinding chain processing, but did not analyze the inner cross section of the grooves. In order to investigate the characteristics of the grooves at any inner cross section of the grooves, the confocal microscopy was used to detect the grooves after laser and laser-grinding chain processing of C/SiC composites. The confocal profiles at any position of C/SiC composite grooves of laser processed and laser-grinding chain processed are, respectively, shown in Figure 15. From Figure 15a, the side walls of the groove were inclined apparently, the maximum depth of the groove was 1824.119 μm, the minimum depth was 1648.307 μm, the average depth was 1738.371 μm, and the depression depth was 175.712 μm; therefore, the groove bottom fluctuated greatly. From Figure 15b, the average depth of the C/SiC composites groove processed by the laser-grinding chain was 2173.138 μm, the fluctuation of the groove bottom could hardly be observed, and only a small inclination between the side wall and the groove bottom. However, the size deviation between the actual depth and the ideal depth still existed, and the side wall was not perpendicular: this was because some errors were unavoidable, such as grinding wheel wear, low process precision, and tool setting errors [48]. Therefore, it can prove the feasibility of laser grinding chain processing of C/SiC composite grooves in terms of size accuracy, shape accuracy, processing efficiency, and surface quality; meanwhile, some errors in chain processing should be adjusted and compensated to further improve the groove quality.

**Figure 16:**
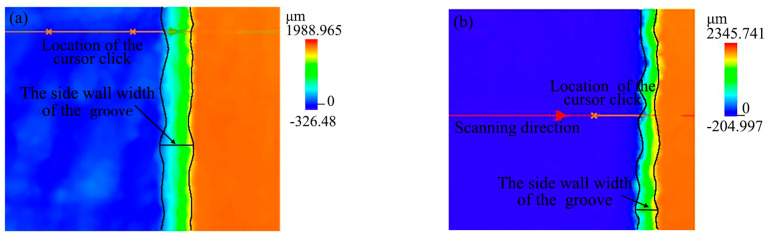
The two-dimensional confocal topography of the groove with different processing methods: (**a**) laser; (**b**) laser-grinding chain.

The two-dimensional confocal topography of the grooves with different processing methods is shown in Figure 16, the color of the grooves both changed from the bottom to the top surface of the grooves followed by dark blue, light blue, green, yellow, and brown. Differently, from the two-dimensional confocal topography, the side wall width of the ablation groove was larger than that of the groove processed by the laser-grinding chain; meanwhile, the color of the ablation groove bottom was relatively complex, while the color of the groove processed by the laser-grinding chain was pure blue without other colors. It indicated that the form accuracy and flatness of the groove were better with the laser-grinding chain process.

## 5. Conclusions

In this paper, in order to improve the processing efficiency and quality of 2.5-dimensional C/SiC composite groove, the laser-grinding chain processing method was proposed, and the laser and grinding parameters in the laser-grinding chain process were optimized by comparing with the surface quality, shape accuracy, and size accuracy based on the optimization experiments. The conclusions are as follows:

1. From the laser processing experiments, with the increase in scanning frequency, the width of the groove top hardly changed, the width of the groove bottom decreased first and then increased, the depth and the taper of the groove increased first and then decreased, the depression depth of the groove bottom decreased first and then increased, and the taper was the smallest when the scanning frequency was 50 kHz. Meanwhile, with the increased scanning spacing, the width of the groove top did not change significantly, the width of the groove bottom and depression depth of the groove decreased first and then increased, and the average depth of the groove increased first and then decreased, and the taper of the groove side wall decreased, and the taper of groove side wall reached the minimum value when the scanning spacing was 25 μm. Moreover, mostly, the taper of the groove’s left side wall was larger than the taper of the groove’s right side wall.
2. From the orthogonal grinding experiments in laser-grinding chain processing C/SiC composite grooves, when the depth of cut was 30 μm, the liner speed of wheel was 62.832 m/min, and the feed speed was 10 mm/min, efficient and precise machining of C/SiC composite grooves could be achieved. Meanwhile, the surface roughness Sa sharply increased with increasing depth of cut, the surface roughness slowly increased with increasing feed rate, and the surface roughness decreased with increasing liner speed of wheel.
3. Taking into account shape accuracy, size accuracy, surface quality, and the influence of these faction the subsequent grinding allowance, the laser power of 80 W, the scanning speed of 300 mm/s, the scanning frequency of 50 kHz, and the scanning spacing of 25 μm were selected to use ablation before grinding, and then, the depth of cut of 30 μm, the liner speed of the wheel of 62.832 m/min, and the feed speed of 10 mm/min were selected for grinding in the laser-grinding chain process
4. Compared with the laser processing C/SiC composite groove, high surface quality, shape accuracy, and size accuracy of the C/SiC composite groove could be obtained by the laser-grinding chain process. While comparing with the grinding C/SiC composite groove, in theory, the high processing efficiency of the C/SiC composite groove could be obtained by the laser-grinding chain process, and the time used for the laser-grinding chain processing groove was about 23/40 of the grinding process.
5. However, the size deviation between the actual depth and the ideal depth still existed, and the side wall was not perpendicular: this was because some errors were unavoidable, such as grinding wheel wear, low process precision, and tool setting errors. Therefore, the adjustment and compensation of errors in chain processing would be the key technology to further improve the groove quality.

## Figures and Tables

**Table 1:** Parameters of nanosecond laser processing.

Parameter	*P* (W)	*v* (mm/s)	*N*	*f* (kHz)	*l*_s_ (um)
Value	80	300	20	50, 100, 150, 200, 250, 300	20
Value	80	300	20	200	5, 10, 15, 20, 25, 30

**Table 2:** Orthogonal design table.

	Depth of Cut *a*_p_ (μm)	Liner Speed of Wheel *v*_s_ (r/min)	Feed Rate *v*_f_ (mm/min)
1	30	37.7	10
2	30	50.3	30
3	30	62.8	20
4	50	37.7	30
5	50	50.3	20
6	50	62.8	10
7	70	37.7	20
8	70	50.3	10
9	70	62.8	30

**Table 3:** Range analysis of the surface roughness *S*_a_.

	Factor A (Depth of Cut)	Factor B (Liner Speed of Wheel)	Factor C (Feed Speed)
K_1_	3.398	5.518	4.419
K_2_	4.189	4.404	4.472
K_3_	5.880	3.545	4.725
k_1_	1.133	1.839	1.473
k_2_	1.396	1.468	1.491
k_3_	1.960	1.182	1.575
R	0.827	0.657	0.102
Priority of factors		A > B > C	
Optimal Scheme		A_1_B_3_C_1_	

## Data Availability

All the data that support the findings of this study are included within the article.
